# New insights into the therapeutic potentials of statins in cancer

**DOI:** 10.3389/fphar.2023.1188926

**Published:** 2023-07-07

**Authors:** Chengyu Liu, Hong Chen, Bicheng Hu, Jiajian Shi, Yuchen Chen, Kun Huang

**Affiliations:** ^1^ Department of Transfusion Medicine, Wuhan Hospital of Traditional Chinese and Western Medicine, Tongji Medical College, Huazhong University of Science and Technology, Wuhan, China; ^2^ Tongji School of Pharmacy, Tongji Medical College and State Key Laboratory for Diagnosis and Treatment of Severe Zoonotic Infectious Diseases, Huazhong University of Science and Technology, Wuhan, China; ^3^ Tongji-RongCheng Biomedical Center, Tongji Medical College, Huazhong University of Science and Technology, Wuhan, China

**Keywords:** statins, cancer, cholesterol, angiogenesis, apoptosis, inflammation

## Abstract

The widespread clinical use of statins has contributed to significant reductions of cardiovascular morbidity and mortality. Increasing preclinical and epidemiological evidences have revealed that dyslipidemia is an important risk factor for carcinogenesis, invasion and metastasis, and that statins as powerful inhibitor of HMG-CoA reductase can exert prevention and intervention effects on cancers, and promote sensitivity to anti-cancer drugs. The anti-cancer mechanisms of statins include not only inhibition of cholesterol biosynthesis, but also their pleiotropic effects in modulating angiogenesis, apoptosis, autophagy, tumor metastasis, and tumor microenvironment. Moreover, recent clinical studies have provided growing insights into the therapeutic potentials of statins and the feasibility of combining statins with other anti-cancer agents. Here, we provide an updated review on the application potential of statins in cancer prevention and treatment and summarize the underneath mechanisms, with focuses on data from clinical studies.

## 1 Introduction

Cancer is the leading cause of death, although much effort has been directed at comprehending carcinogenesis with much progress achieved, effective drug treatment for most cancer types still lack. Dyslipidemia is an important risk factor for carcinogenesis, invasion, and metastasis (Liu et al., 2017a; Quan et al., 2020; Sun et al., 2020; Bian et al., 2021; Lim et al., 2021). Moreover, cancer cells are characterized with increased lipid biosynthesis that meets the metabolic needs of the fast-growing cells and provides cholesterol for membrane formation and stability. In this regard, the anti-cancer properties of lipid-lowering agents have attracted great interest (Matusewicz et al., 2015).

Statins are the most common lipid-lowering drugs, with an estimated 145.8 million users in 2018 (Blais et al., 2021). During recent decades, multiple studies on the anti-cancer effects of statins have been conducted, most of which indicate that statins reduce progression and prolong survival (Matusewicz et al., 2015; Wang et al., 2016; Mei et al., 2017; Chimento et al., 2018; Iarrobino et al., 2018). For examples, a retrospective study conducted on 146,326 women in the United States suggested that statins users had a significantly lower risk of cancer death [hazard ratio (HR), 0.78; 95% CI, 0.71–0.86] compared with never-users (Wang et al., 2016). Another 15-year large-scale observational study of a Danish subgroup including 13 cancers showed that all-cause mortality among patients with cancer who were taking statins was reduced by 15% (95% CI, 13–17) (Nielsen et al., 2012).

**FIGURE 1:**
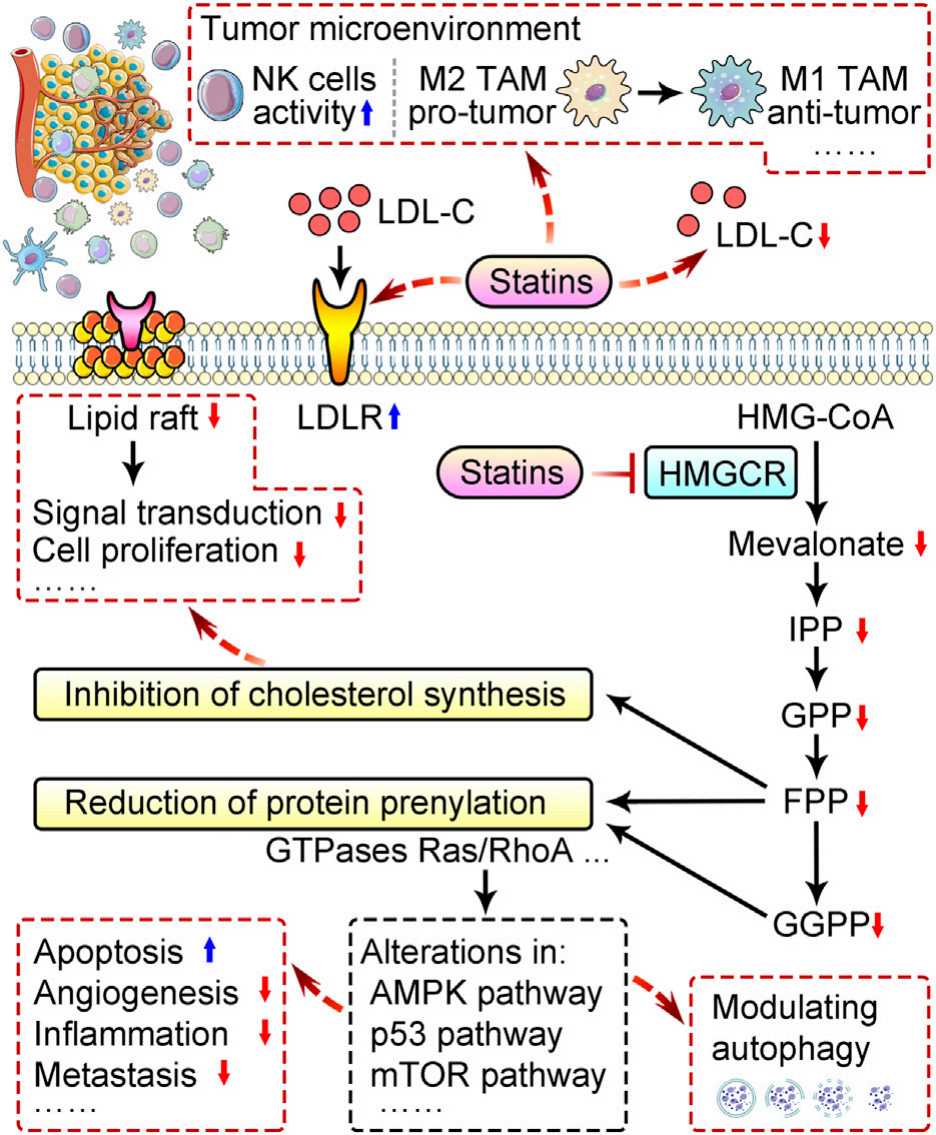
Schematic of mechanisms behind anti-cancer properties of statins. Statins remove serum low-density lipoprotein cholesterol (LDL-C) by upregulating LDL receptor (LDLR) expression in liver and peripheral tissues, and downregulates cholesterol biosynthesis by suppressing mevalonate pathway via inhibition of HMG-CoA reductase (HMGCR). Reduction of cholesterol disrupts the function of lipid rafts and suppresses cancer cell proliferation. Inhibition of the mevalonate pathway by statins also reduces prenylation of proteins like Ras and RhoA GTPases, and subsequently alter multiple pathways to modulate autophagy, promote apoptosis, and suppress angiogenesis, inflammation, metastasis, etc. Statins can also modulate tumor microenvironment via promoting the activity of natural killer (NK) cells, and M2-to-M1 switch, etc. TAM, tumor-associated macrophages; IPP, isopentenyl pyrophosphate; GPP, geranylgeranyl pyrophosphate; FPP, farnesyl pyrophosphate; GGPP, geranylgeranyl pyrophosphate.

As a powerful inhibitor of 3-hydroxy-3-methyl-glutaryl-CoA (HMG-CoA) reductase (HMGCR), statins blocks mevalonate pathway, inhibits *de novo* cholesterol synthesis, and also promotes serum low-density lipoprotein cholesterol (LDL-C) removal by upregulating LDL receptor (LDLR) expression in liver and peripheral tissues (Figure 1) (Stossel, 2008). Reduction of LDL-C hinders cancer progression mainly because rapidly dividing cells require more cholesterol for membrane synthesis (Nielsen et al., 2012; Gobel et al., 2020). Independent of cholesterol-lowering, statins also exhibit pleiotropic effects by downregulating other mevalonate pathway products and disrupting the prenylation of proteins to affect many signaling pathways (Ahmadi et al., 2020; Liu et al., 2020; Jiang et al., 2021; Yang et al., 2023). These cholesterol-independent actions also contribute to the statins’ impacts on growth, apoptosis, autophagy, angiogenesis, inflammation, and metastasis during cancer development (Figure 1) (Ahmadi et al., 2020; Jiang et al., 2021; Liu et al., 2022). Moreover, statins can modulate the tumor microenvironment (Wang et al., 2022a; Qiao et al., 2023) (Figure 1). Based on available pre-clinical and clinical studies, we comprehensively summarize the effects of statins in cancers and relevant mechanisms, and discuss the therapeutic potential and limitations of statin applications in cancer therapy.

## 2 Clinical data of statins in cancers

Despite of some inconsistent results possibly due to cohort diversity and differences in follow-up design, observational studies in the last decade have suggested overall positive impact of statins on clinical outcomes in an array of cancers, including but not limited to colorectal, gastric, breast, lung, liver and kidney cancers (Table 1) (Rosch and McCully, 2013; Wang et al., 2016; Mei et al., 2017; Liu et al., 2019; Tamburrino et al., 2020; Zeng et al., 2023). In a meta-analysis of over 1 million cancer patients, statins use reduces all-cause mortality and cancer-specific mortality by 30% and 40%, respectively (Mei et al., 2017). A recent meta-analysis involving 59,073 patients with hepatocellular carcinoma (HCC) shows that statins use is significantly associated with a reduced risk of HCC development (risk ratio, 0.54; 95% CI: 0.47–0.61) (Islam et al., 2020a). Large-scale observational studies also uncover significant correlation of statins with lower risks of several cancer types including prostate cancer and lymphoma (Table 1) (Graaf et al., 2004; Nielsen et al., 2012; Ren et al., 2021). These results warrant further randomized clinical trials to evaluate subtype-specific effects of statins in cancer prevention and treatment for certain cohorts.

**TABLE 1:** Representative observational studies and interventional clinical studies regarding statins use in cancer.

Large-scale observational studies							
Statins		Cancer type		Study type/patient number		Findings	
Statins		Prostate cancer		Prospective cohort study/44,126		Current statin use was associated with lower risk of PTEN-null and lethal prostate cancer (HR, 0.40; 95% CI, 0.19–0.87; and HR, 0.76; 95% CI, 0.60–0.96; respectively). Allott et al. (2020)	
Statins		Prostate cancer		Case-control study/42,480		The use of statins was associated with a risk reduction of overall prostate cancer (adjusted OR, 0.94; 95% CI, 0.91–0.97) and specifically with advanced prostate cancer (adjusted OR, 0.90; 95% CI, 0.85–0.96). Jespersen et al. (2014)	
Statins (before cancer diagnosis)		Non-Hodgkin lymphoma		Case-control study/18,657		Previous statin administration was associated with a reduced risk of subsequent non-Hodgkin lymphoma (adjusted OR, 0.52; 95% CI, 0.43–0.62). Cho et al. (2015)	
Statins		HCC		Meta-analysis/59,703		Statin use was associated with a reduced risk of HCC development (risk ratio, 0.54; 95% CI, 0.47–0.61) compared with nonusers, supporting the beneficial inhibitory effect of statins on HCC incidence. Islam et al. (2020b)	
Statins		HCC		Meta-analysis/1,774,476		Statin use was associated with reduced HCC risk (HR: 0.52; 95% CI, 0.37–0.72). Zeng et al. (2023)	
Statins (before cancer diagnosis)		Glioblastoma		Prospective cohort study/280,455		Ever statin use (HR, 1.43, 95% CI, 1.10–1.86) was significantly associated with increased glioma risk. Cote et al. (2019)	
Statins (before cancer diagnosis)		Endometrial cancer		Case-control study/77,509		The use of statins was not associated with the risk of endometrial cancer (OR, 1.03; 95% CI, 0.94–1.14). In addition, endometrial cancer risk did not vary substantially with duration or intensity of statin use. Sperling et al. (2017)	
Statins		Colorectal cancer		Meta-analysis/387,518		The use of statins was significantly associated with a decrease in overall mortality (HR, 0.81; 95% CI, 0.76–0.86) and cancer-specific mortality (HR, 0.78; 95% CI, 0.72–0.85) of colorectal cancer. Li et al. (2021)	
Statins (simvastatin being the mostly prescribed lipophilic statin)		Breast cancer		Prospective cohort study/18,769		Simvastatin was associated with a reduced risk of breast cancer recurrence among Danish women diagnosed with stage I–III breast carcinoma (adjusted 10-year risk difference = −0.10, 95% CI, −0.11 to −0.08) Ahern et al. (2011)	
Statins (after cancer diagnosis)		Breast cancer		Retrospective cohort study/17,880		Statin use after a diagnosis of breast cancer reduced mortality due to breast cancer (adjusted HR, 0.84; 95% CI, 0.68–1.04). Cardwell et al. (2015)	
Statins		Gastric cancer		Retrospective cohort study/80,271		Statin use was associated with a reduction of gastric cancer mortality in the general population but not with gastric cancer incidence. Cho et al. (2021)	
Atorvastatin, simvastatin, lovastatin, pravastatin, and rosuvastatin (both pre- and post- cancer diagnosis)		Lung cancer		Retrospective cohort study/19,974		Overall baseline statin exposure was associated with a decrease in mortality risk for squamous-cell carcinoma patients (HR, 0.89; 95% CI, 0.82–0.96) and adenocarcinoma patients (HR, 0.87; 95% CI, 0.82–0.94), but not among those with SCLC. Post-diagnostic statin exposure was associated with prolonged survival in squamous-cell carcinoma patients (HR, 0.68; 95% CI, 0.59–0.79) and adenocarcinoma patients (HR, 0.78; 95% CI, 0.68–0.89). Baseline or post-diagnostic exposure to simvastatin and atorvastatin was associated with extended survival in NSCLC cancer subtypes. Ung et al. (2018)	
Statins		Kidney cancer		Meta-analysis/18,105		Statin use was not significantly associated with PFS (pooled HR 0.92, 95% CI, 0.51–1.65); however, statin use was associated with marked improvements in cancer-specific survival (pooled HR 0.67, 95% CI, 0.47–0.94) and overall survival (pooled HR 0.74, 95% CI, 0.63–0.88) in patients with kidney cancer. Nayan et al. (2017)	
Statins (before cancer diagnosis)		13 cancer types		Retrospective cohort study/295,925		Statin use in patients with cancer was associated with reduced cancer-related mortality. Multivariable-adjusted HR for statin users, as compared with patients who had never used statins, were 0.85 (95% CI, 0.83–0.87) for death from any cause and 0.85 (95% CI, 0.82–0.87) for death from cancer. Nielsen et al. (2012)	
Statins		Not specified		Meta-analysis/1,111,407		Statin use was significantly associated with decreased risk of all-cause mortality (HR, 0.70; 95% CI, 0.66–0.74) compared with nonusers. The observed pooled estimates were retained for cancer-specific mortality (HR, 0.60; 95% CI, 0.47–0.77), PFS (HR, 0.67; 95% CI, 0.56–0.81), recurrence-free survial (HR, 0.74; 95% CI, 0.65–0.83) and disease-free survival (HR, 0.53; 95% Cl, 0.40–0.72). Mei et al. (2017)	
Statins		Not specified		Prospective cohort study/146,326		In a cohort of postmenopausal women, regular use of statins or other lipid-lowering medications was associated with decreased cancer death (HR, 0.78; 95% CI, 0.71–0.86), regardless of the type, duration, or potency of statin medications used. Wang et al. (2016)	
Statins		Not specified		Meta-analysis/175,000		A median of 5 years of statin therapy had no effect on the incidence of, or mortality from, any type of cancer (or the aggregate of all cancer). Cholesterol Treatment Trialists et al. (2012)	

Interventional clinical studies							
Intervention	Cancer type		Mechanisms		Study type/patient number		Findings
Lipophilic statins							
Atorvastatin (80 mg/day before surgery)	Breast cancer		Inhibiting tumor cell growth by downregulation of cyclin D1 and p27		Phase II, non-randomized, window of opportunity trial/50		Atorvastatin treatment in patients with primary invasive breast cancer led to increased protein expression of the tumor suppressor p27, lower cyclin D1 expression, and a decrease in proliferation although not significantly (*p* = 0.08). (NCT00816244) Feldt et al. (2015)
Atorvastatin (10 mg/day)	HCC		NR		Phase IV, double-blind, randomized PC trial/recruiting		No results posted. The aims is to evaluate the effect of atorvastatin for preventing HCC recurrence after curative treatment. The primary endpoint is to compare the 3-year cumulative incidence of recurrent HCC between the intervention group and control counterpart. (NCT03024684)
Atorvastatin (20 mg/day) plus zoledronate	Kidney cancer		Bisphosphonates and statins target different steps in the mevalonate pathway, providing a synergistic effect		Phase II, single-arm, pilot trial/11		The combination use of zoledronate and atorvastatin (or fluvastatin) were well tolerated, affected certain bone biomarkers and provided bone response. (NCT00490698) Manoukian et al. (2011)
Atorvastatin (Before surgery) plus metformin	Breast cancer		Inhibiting signaling pathways including PI3K/Akt/mTOR and AMPK		Phase I, window of opportunity trial/23		No results posted. The aim is to assess whether tumor proliferation is reduced following ∼2 weeks of treatment with metformin plus atorvastatin in patients with newly diagnosed breast cancer. (NCT01980823)
Atorvastatin (40 mg/day) plus radiotherapy and temozolomide	Glioblastoma		NR		Phase II, single-arm trial/36		80% of patients discontinued because of disease progression. High LDL level was an important independent predictor of poor cancer-related outcome. (NCT02029573) Altwairgi FA et al. (2019)
Simvastatin (40 mg/day)	Ovarian cancer		NR		Phase I, single-arm, pilot trial/recruiting		No results posted. The aim is to evaluate the feasibility and effects of simvastatin to reduce cancer progression among patients with platinum-sensitive ovarian cancer treated with carboplatin and liposomal doxorubicin. (NCT04457089)
Simvastatin (40 mg/day) plus fluorouracil, adriamycin, and cyclophosphamide (FAC)	Locally advanced breast cancer		Inducing apoptosis and inhibiting tumor cell growth		Phase II, double-blind, randomized PC trial/70		Simvastatin combined with FAC showed improvements in ORR and pathological response in patients with locally advanced breast cancer. Although no statistically significant difference was documented, there was a trend for better activity and tolerability. (NCT04418089) Yulian et al. (2021)
Simvastatin (80 mg/day) plus XELOX and bevacizumab	Metastatic colorectal cancer		Inducing tumor cell senescence and apoptosis, and showing anti-angiogenesis effect		Phase II, single-arm trial/60		Addition of simvastatin to XELOX and bevacizumab showed comparable clinical efficacy in patients with metastatic colorectal cancer as first-line chemotherapy and did not increase toxicity. The median PFS was 10.4 months, the disease-control rate and overall RR was 88.3% and 58.3%. (NCT02026583) Kim et al. (2019)
Simvastatin (40 mg/day) plus gefitinib	NSCLC		Impairment of protein prenylation and interference with lipid rafts both affect the function of EGFR and EGFR signaling		Phase II, randomized trial/106		The combination of simvastatin and gefitinib resulted in higher RR (40% vs. 0%, *p* = 0.043) and longer PFS (3.6 months vs. 1.7 months, *p* = 0.027) compared with gefitinib alone in subgroup of patients with wildtype EGFR non-adenocarcinomas. (NCT00452244) Han et al. (2011)
Simvastatin (40 mg/day) plus afatinib	NSCLC		Inhibition of RAS activation and downstream signaling cascades		Phase II, randomized trial/68		Combination of simvastatin plus afatinib was well-tolerated, but did not improve RR and PFS compared with afatinib alone in patients with advanced non-adenocarcinomas who progressed after chemotherapy regimens. (NCT01156545) Lee et al. (2017)
Simvastatin (40 mg/day) plus capecitabine and cisplatin	Gastric cancer		Regulating modifications of Ras and RhoA, inducing apoptosis, and lowering VEGF serum levels		Phase III, double-blind, randomized PC trial/244		Addition of simvastatin to capecitabine-cisplatin did not increase PFS in patients with previously untreated advanced gastric cancer, although it did not increase toxicity. (NCT01099085) Kim et al. (2014)
Simvastatin (40 mg/day) plus gemcitabine	Pancreatic cancer		Impairment of protein prenylation and interference with lipid rafts both affect the function of EGFR and EGFR signaling		Phase II, double-blind, randomized PC trial/114		Addition of simvastatin to gemcitabine in advanced pancreatic cancer did not provide clinical benefit, although it did not result in increased toxicity. The median time to progression was not significantly different between the two arms (2.4 months vs. 3.6 months, *p* = 0.903). (NCT00944463) Hong et al. (2014)
Simvastatin (40 mg/day) plus FOLFIRI/XELIRI chemotherapy regimens	Metastatic colorectal cancer		Impairment of protein prenylation and intracellular signal transduction		Phase III, double-blind, randomized PC trial/269		Addition of simvastatin to the regimens did not improve median PFS (5.9 months vs. 7.0 months, *p* = 0.826) in patients with previously treated metastatic colorectal cancer nor did it increase toxicity. (NCT01238094) Lim et al. (2015)
Simvastatin (40 mg/day) plus chemotherapy/radiation	Rectal cancer		NR		Phase II, double-blind, randomized PC trial/222		No results posted. The primary objective is rates of favorable MRI-based tumor regression grading. Patients receive simvastatin or placebo daily for 90 days starting 1 week prior to standard preoperative chemoradiotherapy. (ACTRN 12617001087347) Jameson et al. (2019)
Simvastatin (80 mg/day) plus capecitabine	Locally advanced rectal cancer		NR		Phase II, single-arm trial/60		No results posted. The aim is to investigate the synergistic effect of simvastatin combined with capecitabine and radiotherapy in locally advanced rectal cancer patients. The primary outcome is pathologic complete response rate. (NCT02161822)
Simvastatin (20 mg/day before surgery) plus metformin	Bladder cancer		Inhibiting signaling pathways including PI3K/Akt/mTOR and AMPK		Phase II, single-arm, window of opportunity trial/44		No results posted. The aim is to evaluate the effect and feasibility of using a combination of metformin and simvastatin as a neoadjuvant treatment for patients with invasive bladder cancer who are to undergo cystectomy. (NCT02360618)
Lovastatin (0.5–2 mg/kg) plus thalidomide and dexamethasone	Multiple myeoloma		Anti-neoplasmatic property and inducing apoptosis		Randomized trial/91		Lovastatin plus thalidomide–dexamethasone prolonged OS and PFS compared with thalidomide–dexamethasone alone in patients with relapsed or refractory multiple myeloma. Hus et al. (2011)
Fluvastatin (80 mg/day)	Localized prostate cancer		Inducing tumor cell apoptosis		Phase II, single-arm, window of opportunity, pilot trial/33		A median 2.7-fold increase in cleaved Caspase-3 positivity (95% CI: 1.9–5.0, *p* = 0.007) was observed in post-fluvastatin RP tissues compared with matched pre-treatment biopsy controls. Fluvastatin was associated with promising effects on tumor cell apoptosis. Longo et al. (2020)
Fluvastatin (80 or 20 mg/day)	Breast cancer		Inducing apoptosis and suppressing tumor cell proliferation		Randomized, peri-operative window trial/40		Administration of fluvastatin for 3–6 weeks before surgery decreased proliferation of high-grade tumors by a median of 7.2% (*p* = 0.008), and increased apoptosis in 60% of high-grade tumors; for low-grade tumors, these effects were less evident. Garwood et al. (2010)
Fluvastatin (80 mg/day)	Breast cancer		NR		Single-arm, non-randomized, biomarker modulation trial/30		Lovastatin was technically feasible and generally well-tolerated in women at increased risk of developing breast cancer, but no significant biomarker modulation was observed. The results did not exclude a favorable effect on breast cancer risk. (NCT00285857) Vinayak et al., (2013)
Hydrophilic statins							
Rosuvastatin (40 mg/day) plus chemoradiation therapy	Rectal cancer		Sensitizing cancer tissues and protects normal tissues to the effects of radiation		Phase II, single-arm trial/45		No results posted. The aim is to evaluate whether the addition of rosuvastatin to standard chemoradiation therapy for the treatment of locally advanced rectal cancer may improve the pathological response rate and survival compared to standard chemoradiation therapy alone. Rosuvastatin treatment starts 2 weeks prior to the initiation of radiation at week 1 and stops 4 weeks after the completion of radiation. (NCT02569645)
Pravastatin (40 mg/day) combined with etoposide plus cisplatin or carboplatin	SCLC		NR		Phase III, double-blind, randomized PC trial/846		Pravastatin plus standard chemotherapy did not offer additional benefit compared with chemotherapy alone. The median PFS was 7.7 months vs. 7.3 months. The median OS (pravastatin v placebo) was 14.6 months in both groups for limited disease and 9.1 months versus 8.8 months, respectively, for extensive disease. (NCT00433498) Seckl et al. (2017)
Pravastatin (40 mg/day) plus chemotherapy	Gastric carcinoma		NR		Phase II, randomized trial/30		Addition of pravastatin to epirubicin/cisplatin/capecitabine did not improve progression-free rate at 6 months, RR, PFS and OS. Konings et al. (2010)
Pravastatin (40 mg/day) plus TAE and 5-FU	HCC		NR		Randomized trial/91		Pravastatin prolonged the survival of patients with advanced HCC (median survival, pravastatin group vs. controls, 18 months vs. 9 months, *p* = 0.006). Kawata et al. (2001)
Pravastatin (40 mg/day) plus sorafenib	HCC		Inhibiting Raf-Ras -MAPK pathway. The anti-invasive and anti-metastatic action of pravastatin is a complement to the anti-angiogenic action of sorafenib		Phase III, randomized trial/312		Addition of pravastatin to sorafenib did not improve survival in patients with advanced HCC, with no difference in median OS between sorafenib-pravastatin and sorafenib groups (10.7 months vs*.* 10.5 months; HR = 1.00; *p* = 0.975). (NCT01075555) Jouve et al. (2019)
Pravastatin (40 mg/day)	Esophageal cancer and stomach cancer		NR		Phase IV, randomized trial/recruiting		No results posted. The objective is to evaluate the efficacy of treatment (increase in survival and recurrence-free period of the disease) with pravastatin in patients with advanced esophageal cancer and stomach cancer. The experimental group will receive pravastatin orally daily for 2 years. (NCT01038154)

Abbreviations: HCC, hepatocellular carcinoma; HR, hazard ratio; LDL, low density lipoprotein; NR, not reported; NSCLC, non-small cell lung cancer; OR, odds ratio; ORR, objective response rate; OS, overall survival; PC, placebo-controlled; PFS, progression-free survival; RR, response rate; SCLC, small cell lung cancer; TAE, transcatheter arterial embolization.

Various interventional clinical trials regarding anti-cancer ability of statins are ongoing, either given alone or in combination, with some already posted positive results (Table 1). A perioperative window trial in women with stage 0/1 breast cancer demonstrated that administration of fluvastatin for 3–6 weeks before surgery decreased proliferation of high-grade tumors by a median of 7.2% (*p* = 0.008), and increased apoptosis in 60% of high-grade tumors; while for low-grade tumors, these effects were less evident (Garwood et al., 2010). Similarly, 2-week atorvastatin treatment before surgery decreased tumor cell proliferation in patients with primary invasive breast cancer (Feldt et al., 2015). However, clinical trials applied statins in combination with other anti-cancer drugs gave less satisfactory results. A recent phase III trial found adding pravastatin to sorafenib did not improve survival in patients with advanced HCC, with no difference in median overall survival between sorafenib-pravastatin and sorafenib groups (10.7 months vs*.* 10.5 months; HR, 1.00; *p* = 0.975) (Jouve et al., 2019). Use of simvastatin in combination with chemotherapy drugs fail to benefit patients in most trials, except when combined with fluorouracil, adriamycin, and cyclophosphamide to treat patients with locally advanced rectal cancer (Table 1) (Yulian et al., 2021). How to take advantage of statins to promote current anti-cancer therapy remains a serious question awaiting in-depth mechanistic studies.

## 3 Mechanisms behind statins’ anti-cancer effects

The anti-cancer effect of statins are closely related to their inhibitory effect on HMG-CoA reductase and mevalonate pathway. Statins-mediated reduction of cholesterol leads to interruption of cell membrane structure and cholesterol-related biological function (Figure 1). Statins also downregulate non-cholesterol products of mevalonate pathway, including isopentenyl pyrophosphate (IPP), farnesyl pyrophosphate (FPP), and geranylgeranyl pyrophosphate (GPP), thereby suppress prenylation of proteins like small monomeric GTPases, primarily Ras and RhoA proteins, and consequently alter multiple cancer pathways (Figure 1) (Gobel et al., 2020; Jiang et al., 2021). Here, we introduce how statins exhibit tumor-suppressing activity by downregulating cholesterol, and how statins regulate multiple aspects including angiogenesis, apoptosis, autophagy, metastasis, tumor microenvironment and drug resistance in cancer.

### 3.1 Downregulating cholesterol

Cholesterol, the ubiquitous precursor to sterol hormones, is one of the basic building elements of cell membranes. Moreover, cholesterol regulate multiple signaling pathways involved in tumorigenesis and progression. Its endogenous synthesis is catalyzed by HMGCR (Mullen et al., 2016), while its uptake is regulated by LDLR. High intracellular cholesterol in normal cells blocks HMGCR mediated cholesterol biosynthesis and upregulates LXR α/β mediated cholesterol efflux transporter expression. In cancer cells, the presence of intracellular cholesterol does not affect cholesterol biosynthesis and uptake. The highly active cholesterol metabolism within cancer cells facilitates tumor progression and thus becomes a vulnerability that may be targeted (Mehta et al., 1998; Mullen et al., 2016; Zhou et al., 2018; Huang et al., 2020). Additionally, in the tumor microenvironment (TME), cholesterol metabolism is generally enhanced; thus targeting cholesterol synthesis can also modulate TME (Huang et al., 2020; Zhu et al., 2021). Indeed, in lung cancer cells, simvastatin remodels TME and reverses epithelial-mesenchymal transition (EMT) by re-polarizing tumor-associated macrophages (TAMs) from M2 to M1 via cholesterol-associated LXR/ABCA1 regulation (Jin et al., 2019).

Lipid raft, a specialized cholesterol-rich region of the cell membrane, facilitates membrane-initiated signaling events through compartmentalization of signaling pathways (Simons and Ikonen, 1997; Boudreau et al., 2010). Importantly, lipid raft is a key player in statin-mediated inhibition of tumor growth and migration (Simons and Ikonen, 1997; Boudreau et al., 2010; Yang et al., 2023). Simvastatin treatment reduced tumor cell growth, cellular cholesterol levels, cholesterol content in lipid rafts and membrane integrity (Zhuang et al., 2005; Menter et al., 2011). On the other hand, elevation of circulating cholesterol by cholesterol-enriched diet promoted tumor growth in a xenograft mouse model for prostate cancer (Zhuang et al., 2005). Disruption of lipid rafts by simvastatin also re-sensitized paclitaxel resistance in lung cancer cells by suppressing integrin-β3/FAK signaling pathway and focal adhesion formation (Jin et al., 2019). Moreover, in myeloproliferative neoplasms (MPN), aberrant JAK2 signaling plays a crucial tumor-promoting role, while JAK inhibitors did not induce patient remission; alternatively, simvastatin, lovastatin and atorvastatin inhibited mutated JAK2 localization to lipid rafts, consequently inhibited JAK2-V617-dependent growth and induced apoptosis in MPN cells, and suppressed primary erythroid colony formation of primary cells from MPN patients (Griner et al., 2013). These studies unequivocally suggest that statin-induced reduction on cholesterol alters signaling transduction to interfere with cell proliferation and metastasis, while the exact molecular alteration behind statin-induced changes of lipid raft remain not completely defined.

### 3.2 Inhibiting angiogenesis

Angiogenesis, formation of new blood vessels from pre-existing vessels, is an important event in cancer growth and hematogenous metastasis (Zhang et al., 2022; Xiong et al., 2023). Inhibition of angiogenesis with several FDA-approved inhibitors has been an established therapeutic strategy for many solid tumors (Chen et al., 2018; Li et al., 2019; Liu et al., 2019; Zhang et al., 2022). Anti-angiogenic effect of statins has attracted growing attention (Weis et al., 2002; Dulak and Jozkowicz, 2005; Zahedipour et al., 2022). The anti-angiogenic effect of cerivastatin is cholesterol-independently achieved by inhibiting the RhoA/focal adhesion kinase/AKT pathways (Vincent et al., 2001). Similarly, simvastatin interferes with angiogenesis by inhibiting RhoA geranylgeranylation (Park et al., 2002). Powerful anti-angiogenic effect of atorvastatin was evident in glioblastoma 3D spheroids by downregulating expression of VEGF and CD31 (Bayat et al., 2018), and reduction of angiogenesis by rosuvastatin was observed in tumor-bearing mice (Weis et al., 2002). Importantly, simvastatin potentiated the anti-angiogenic effects of bevacizumab on human colorectal cancer cells (Lee et al., 2014), and addition of simvastatin to XELOX and bevacizumab showed comparable clinical efficacy (disease-control rate, 88.3%) in patients with metastatic colorectal cancer with a favorable safety profile in a phase II study (Kim et al., 2019).

### 3.3 Inducing apoptosis

Statins induce cell apoptosis in different cancer types including lung, prostate, colorectal, and breast cancers (Zaleska et al., 2018; Ahmadi et al., 2020; Juarez and Fruman, 2021; Guo et al., 2022); with a tendency to induce greater-degree of apoptosis in malignant cells than in non-malignant ones (Wong et al., 2002; Wu et al., 2004). This is possibly due to enhanced dependency of malignant cells on signaling pathways including AMPK, AKT, mTOR, and p53 pathways, and autophagy pathway (Chou et al., 2019; Wang et al., 2022b). AMPK is a cellular energy sensor that inhibits cell proliferation and induces cancer cell apoptosis, and statins can activate AMPK pathway; moreover, statins-associated AMPK activation led to decreased lipid accumulation in liver which may decrease risk to liver cancer (Misirkic et al., 2012; Dehnavi et al., 2021). In glioma cell lines, simvastatin induced apoptosis by inhibiting AKT activation and mTOR pathways (Misirkic et al., 2012; Dehnavi et al., 2021). In lung adenocarcinoma, simvastatin enhanced caspase-dependent apoptotic progress by promoting mutant p53 protein degradation (Chou et al., 2019). Additionally, in small cell lung cancer, statins induced oxidative stress accumulation and apoptosis through suppressing the geranylgeranyl diphosphate (GGPP) synthase 1 (GGPS1)-RAB7A-autophagy axis, overcame both intrinsic and acquired chemoresistance *in vivo* across PDX models bearing high GGPS1 levels (Guo et al., 2022). The capacity of modulating apoptosis makes statins promising candidates for anti-cancer treatment.

### 3.4 Modulating autophagy

Autophagy plays a dual role in cancer, as either a promoter or a suppressor (Ashrafizadeh et al., 2020; Mengual et al., 2022). On one hand, statins can induce apoptosis via inhibiting autophagy (Chou et al., 2019; Guo et al., 2022); on the other hand, statins can suppress cancer cell viability via inducing autophagy in multiple cancers, such as ovarian cancer, lung adenocarcinoma, malignant pleural mesothelioma, melanoma, and pancreatic cancer (Ashrafizadeh et al., 2020; Mengual et al., 2022). Several signaling pathways have been implicated in the regulation of statin-mediated autophagy, including the mevalonate pathway, AMPK/mTOR pathway, and the nuclear accumulation of p53 (Yang and Chen, 2011; Zhang et al., 2012). For examples, fluvastatin reduced breast cancer cell viability by activating AMPK-mTOR dependent autophagy activation (Elimam et al., 2020), and prevented lung adenocarcinoma bone metastasis in nude mice via inducing autophagy that triggered by increased nuclear p53 expression (Yang et al., 2017). Moreover, the suppressive effect of lovastatin on primary tumors and metastasis in malignant mesothelioma was due to mTOR-independent induction of autophagic changes (Asakura et al., 2011). In lymphoma cells, fluvastatin treatment induced autophagy contributed to fluvastatin-induced apoptosis, which can be blocked by metabolic products of the HMG-CoA reductase reaction (Qi et al., 2013). However, in HCC and colorectal carcinoma cells, atorvastatin inhibited cell growth via inducing apoptosis, while promoted cell survival via inducing autophagy by activating AMPK/p21-dependent endoplasmic reticulum stress response (Yang et al., 2010). The mixed results in preclinical studies suggest that a refined classification needs to be considered when investigating the autophagy-related impacts of different statins in different cancer types. The combination treatment of statins and autophagic inhibitors in cancer therapy also warrants intensive investigation.

### 3.5 Reducing risks of metastasis

Metastasis is a major cause of cancer-related death. Take prostate cancer (PC) as an example, localized PC is frequently curable, while treatment for metastatic PC is challenging with limited therapeutic options and inevitable drug resistance (Scheinberg et al., 2023). Accumulating studies have suggested that circulating lipids were associated with PC aggressiveness and PC death, and that statin use was associated with reduced risks of metastatic PC and PC mortality (Raval et al., 2016; Van Rompay et al., 2019; Scheinberg et al., 2023). According to a large population-based cohort study with 25-year follow-up data, statins reduced the risk of aggressive PC (HR 0.52, 95% CI: 0.40–0.68), and statin users had a 49% lower risk of PC mortality (HR 0.51, 95% CI: 0.41–0.63) (Van Rompay et al., 2019). Similarly, a meta-analysis of 34 studies (including prospective randomized clinical trials and observational studies) showed that statins use was associated with over 20% reduction in the risks of both PC metastases (pooled HR 0.78, 95% CI: 0.68–0.87) and PC mortality (pooled HR 0.76, 95% CI: 0.63–0.91) (Raval et al., 2016). Moreover, *in vivo* studies found that simvastatin prevented the skeletal metastasis of breast cancer by inhibiting the expression of cancer stem cell marker CD44 and enhancing the expression of p53 (Mandal et al., 2011). Pravastatin reduced the lung metastasis of rat hepatocellular carcinoma by downregulating the expression and activity of liver matrix metalloproteinase-9 (Taras et al., 2007).

### 3.6 Modulating tumor microenvironment

Recent studies have demonstrated that tumor microenvironment (TME), which is characterized by metabolic reprogramming and hypoxia, play important roles in tumor progression (Chen et al., 2018; Liu et al., 2019). Cholesterol metabolism in TME is generally enhanced, as evidenced by increased cholesterol biosynthesis and uptake. In situations in which lipids and/or oxygen is limited, such as in the glioblastoma microenvironment, the master transcription factor SREBP2 and its downstream targets, including mevalonate-pathway enzymes are significantly upregulated in tumor (Lewis et al., 2015). Beyond SREBP2, another transcription factor, RORγ, which activates the cholesterol-biosynthesis program, is upregulated in triple-negative breast cancer and facilitates tumor progression (Cai et al., 2019). In addition to enhanced *de novo* cholesterol synthesis, increasing cholesterol uptake is observed in cancer cells. An extreme example is that some anaplastic large cell lymphoma cells express increased levels of LDLR and fully rely on cholesterol uptake to acquire exogenous cholesterol, thus supporting proliferation (Garcia-Bermudez et al., 2019). Moreover, another group of cholesterol metabolites, cholesteryl esters (CE) and oxysterols, are enriched in TME; accumulation of CE and oxysterols is another common signature in cancer (Li et al., 2016; Kloudova et al., 2017). Thus statins can regulate the metabolic TME due to its impact on multiple metabolic pathways (Chen et al., 2020; Huang et al., 2020; Liu et al., 2020; Zhu et al., 2021; Yang et al., 2023). For examples, simvastatin re-polarized TAMs, promoted M2-to-M1 phenotype switch, and suppressed epithelial-mesenchymal transition in lung cancer via cholesterol-associated LXR/ABCA1 regulation (Jin et al., 2019). Statins also downregulate the mevalonate-pathway product coenzyme Q (CoQ) and lead to severe oxidative stress, resulting in significant ROS production, which helps to improve the efficacy of chemotherapy (McGregor et al., 2020). Fatty acid synthesis increases along with the accumulation of H^+^, which contributes to the generation of acidic TME; while statins significantly reduced plasma free fatty acid concentrations (Sorrentino et al., 2014; Sahebkar et al., 2016; Chen et al., 2020; Liu et al., 2020; Yang et al., 2023). Pre-treatment of simvastatin reduces lactate content in head and neck tumors, and promotes tumor sensitivity to monocarboxylate transporter 1 (MCT1) inhibitors (Mehibel et al., 2018).

In addition, statins can alter the gene expression mediated by HIF-1α, a key regulator for hypoxia response, by stimulating HIF-1α ubiquitin/proteasome degradation (Hisada et al., 2012). In breast cancer, simvastatin-mediated activation of AMPK suppressed breast tumor angiogenesis by blocking HIF-1α (Fukamachi et al., 2013; Wang et al., 2018; Jin et al., 2019). Moreover, the anti-tumor effects of statins were associated with their effect on a variety of immune cells in TME other than TAMs, such as lymphocytes and natural killer cells (NK cells) (Wang et al., 2022a; Qiao et al., 2023). For examples, the combination of statins and difluoromethylornithine (DFMO) significantly suppressed colon cancer by increasing the activity of functional NK cells (Janakiram et al., 2016). Moreover, statins treatment induced MHC class I Chain-related protein A overexpression and sensitized tumor cells to lysis by NK cells (Pich et al., 2013). Whether these effects of statins can be adapted in improving anti-cancer immunotherapy awaits further experimental and clinical exploration.

### 3.7 Overcoming drug resistance

Cancer resistance, which is characterized by tumor relapse or spread, remains a major challenge in clinical oncology (Kartal-Yandim et al., 2016; Quan et al., 2020). A range of studies have reported the effects of statins on overcoming the resistance to various anti-cancer drugs (Tilija Pun and Jeong, 2021). For examples, simvastatin effectively improved doxorubicin cytotoxicity in human malignant mesothelioma cells (Riganti et al., 2006). In chronic lymphocytic leukemia, activation of RhoA/RhoA kinases, Ras/ERK1-2, Akt, HIF-1α, and P-glycoprotein protected cells from doxorubicin; while simvastatin inhibited these effects and sensitized cells to doxorubicin (Rigoni et al., 2015). Combined treatment of simvastatin 5-fluorouracil (5-FU) synergistically suppressed colon tumors *in vivo* by inhibiting inflammation, angiogenesis, and metastasis (Luput et al., 2020). In addition, chemo-resistant small cell lung cancer xenograft showed dependence on mevalonate-GGPP pathway, which can be suppressed by statins (Guo et al., 2022). Apart from chemotherapeutic drugs, statins also contributed to overcoming the resistance to targeted drugs including the widely applied EGFR tyrosine kinase inhibitor gefitinib. Addition of simvastatin to gefitinib enhanced apoptosis in gefitinib-resistant EGFR T790M mutant NSCLC cells by suppressing the activation of AKT and β-catenin/survivin (Hwang et al., 2014). Moreover, atorvastatin reversed KRAS-mediated gefitinib resistance in NSCLC cells by inhibiting HMG-CoA reductase-dependent disruption of Kras/Raf and Kras/PI3K complexes (Chen et al., 2013). There are ongoing trials of statins use combined with other anti-cancer agents in different cancers including NSCLC, SCLC, HCC, gastric cancer, locally advanced breast cancer, metastatic colorectal cancer, etc. (Table 1)

## 4 Conclusion and future perspectives

Accumulating pre-clinical and clinical trials of statins in different cancers suggested overall beneficial role of statins with a favorable safety profile in cancer treatment and prevention. The anti-cancer effects, as well as their well-tolerance, low cost, and much lower toxicity compared with the conventional chemotherapy drugs, attract increasing consideration of repurposing statins as a promising strategy for cancer treatments.

Beyond *de novo* cholesterol biosynthesis, most cells can acquire cholesterol via uptake extracellular cholesterol by various molecules including LDLR. Therefore, cancer cells may bypass their dependency on *de novo* cholesterol biosynthesis by relying on exogenous cholesterol, such as LDL/HDL, which limits the anti-cancer effect of statin treatment. Inhibition of cholesterol uptake has shown anti-cancer property in some cases, for examples, using shRNA for LDLR increases the efficacy of gemcitabine in pancreatic cancer (Guillaumond et al., 2015); an FDA approved cholesterol uptake blocker ezetimibe retards *in vivo* prostate cancer progression by inhibiting angiogenesis (Solomon et al., 2009). Therefore, combination of statins and cholesterol uptake blocker may provide enhanced anti-cancer effect, which warrants more in-depth studies. It is currently difficult to predict the type of cancers that particularly sensitive to statin therapy. However, encouraging results from some trials (Garwood et al., 2010; Bjarnadottir et al., 2013; Harshman et al., 2015) suggest that patients with hormone-dependent cancers, such as breast cancer and prostate cancer, may benefit from adding statins to their treatment. This may be partly because cholesterol is the precursor of hormones such as oestrogen and androgens, which have a major role in the development of these cancers (Finlay-Schultz and Sartorius, 2015). Clinical trials are required to further define the subset of cancers that are more statin-sensitive (Mullen et al., 2016).

The heterogeneous physiological effects of different types of statins in different cancer types need to be considered. Depending on chemical structure, statins are classified as either lipophilic or hydrophilic (Istvan and Deisenhofer, 2001; Sirtori, 2014). Some studies suggested stronger association of lipophilic statins than hydrophilic ones with lower cancer-specific mortality (Liu et al., 2017b; Majidi et al., 2021). A plausible reason is that compared with hydrophilic statins, lipophilic statins have higher pro-apoptotic activity, and a greater ability to penetrate cell membrane and enter cells through passive diffusion (Hamelin and Turgeon, 1998; Dulak and Jozkowicz, 2005; Kato et al., 2010; Menter et al., 2011), while further investigations are warranted.

Among many explanations of anti-cancer effects of statins, the cholesterol-dependent function has been comprehensively-characterized, while the cholesterol-independent impacts are relatively less studied. Many questions remain to be explored, such as determination of proper dosage of statins to avoid biphasic effects, whether statins can be applied in combination with anti-cancer drugs to improve therapy, etc. Improved understanding of relevant molecular mechanisms will help elucidating the anti-cancer properties of statins and guide future clinical trials.
